# Phonon‐Modulated Kerr Nonlinearity in Ultrathin 2*H*‐MoTe_2_

**DOI:** 10.1002/smsc.70424

**Published:** 2026-10-14

**Authors:** Shaoxiang Sheng, Yang Luo, Chenyu Wang, Sayooj Sateesh, Yaxian Wang, Marko Burkhard, Sayantan Patra, Bhumika Chauhan, Ashish Arora, Sheng Meng, Manish Garg

**Affiliations:** ^1^ Max Planck Institute for Solid State Research Stuttgart Germany; ^2^ Tsientang Institute for Advanced Study Hangzhou China; ^3^ Hefei National Laboratory University of Science and Technology of China Hefei China; ^4^ Beijing National Laboratory for Condensed Matter Physics and Institute of Physics Chinese Academy of Sciences Beijing China; ^5^ School of Physical Sciences University of Chinese Academy of Sciences Beijing China; ^6^ Indian Institute of Science Education and Research Pune India

**Keywords:** 2*H*‐MoTe_2_, coherent phonons, cross‐phase modulation, dual‐pump control, Kerr nonlinearity

## Abstract

Controlling nonequilibrium responses in optically driven quantum materials is essential for advancing applications in energy conversion, ultrafast electronics, and quantum computation. Nonlinear optical spectroscopy serves as a powerful tool to investigate ultrafast electron and phonon dynamics in these systems; however, conventional nonlinear approaches often require intense laser pulses and typically encounter a strong background. Here, we introduce a phase‐sensitive nonlinear spectroscopic technique that operates at low pulse energies (~10 pJ) and enables real‐time monitoring and active control of coherent phonons in few‐layer (three to five layers thick) 2*H*‐MoTe_2_. Upon excitation with ultrashort (~10 fs) pump pulses, we achieve displacive excitation of coherent phonons, which periodically modulate the Kerr nonlinearity of the material, leading to cross‐phase modulation (XPM) of a delayed probe pulse. This phase modulation induces spectral broadening and oscillations in the center of mass (COM) of the probe spectrum in time, enabling the detection of subtle nonlinear optical responses in a background‐free manner. The nonlinear response can be selectively amplified or attenuated by adjusting the strength of the pump pulse. A dual‐pump pulse scheme enables precise control of phonon oscillations, allowing selective activation or suppression of specific phonon modes and correspondingly the modulation of the Kerr nonlinearity.

## Graphical abstract

Phase‐sensitive cross‐phase modulation (XPM) encodes the pump‐induced transient nonlinear optical response of ultrathin 2*H*‐MoTe_2_ into the time‐dependent phase of a delayed broadband probe pulse. Dual‐pump excitation enables coherent control of the phonon‐modulated nonlinear response. The resulting spectral redistribution and center‐of‐mass (COM) shift provide a sensitive readout of the coupled ultrafast electronic and coherent‐lattice dynamics.

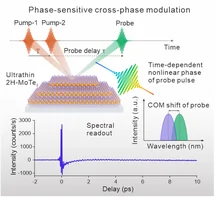

## 1 Introduction

Ultrafast optical control of quantum materials provides a powerful approach to access nonequilibrium states of matter [1, 2] such as light‐induced superconductivity [3, 4], magnetism [5], and topological phases [6, 7]. Understanding these transient phenomena requires insights into the complex interplay between electronic excitations and coherent phonon motions, yet state‐of‐the‐art techniques struggle to disentangle these dynamics at their natural timescales while providing phase‐sensitive information.

Nonlinear optical processes, e.g., the Kerr effect—a third‐order nonlinear (*χ* ^(3)^) response leading to intensity‐dependent modulation of the refractive index—are crucial in ultrafast signal processing and the investigation of complex light‐matter interactions [8, 9, 10, 11]. In particular, cross‐phase modulation (XPM), where a “pump” pulse‐induced nonlinearity modulates the phase of a time‐delayed “probe” pulse via the nonlinear Kerr effect, offers a highly phase‐sensitive approach for tracking both coherent and incoherent quasiparticle dynamics [12]. In contrast to self‐phase modulation (SPM), which entangles various nonlinear interactions within the material [13], XPM provides a phase‐locked detection of dynamics evolving in real‐time. In this process, the intensity, polarization, and spectra phase or frequency of the probe pulse can be dynamically modulated via interactions with the material pre‐excited by an ultrafast pump pulse [14, 15, 16].

Leveraging XPM for ultrafast material control is particularly compelling in layered 2D materials, such as graphene and related materials and transition metal dichalcogenides (TMDCs), which exhibit pronounced Kerr nonlinearities alongside exceptional optical and electronic properties [17, 18, 19, 20, 21, 22]. These materials have been integrated with dielectric waveguides, resonators, and photonic circuits to enhance nonlinear optical processes, including SPM, four‐wave mixing (FWM), and frequency conversion, highlighting their potential for compact and actively tunable nonlinear photonic devices [18, 19, 20]. Among them, MoTe_2_ is distinguished by its rich nonequilibrium states and structural phase transitions under intense optical pulse excitation [23, 24, 25, 26], making it a promising platform for all‐optical modulation and control.

## 2 Phonon‐Mediated Cross‐Phase Modulation

In this work, we introduce a phase‐sensitive XPM spectroscopic technique that directly resolves and controls femtosecond electron and phonon dynamics in few‐layer 2*H*‐MoTe_2_. Ultrashort laser pulses generated by an ultrabroadband Ti:sapphire oscillator were split into two spectrally separated “pump” and “probe” pulses using an 800 nm shortpass dichroic mirror (DMSP) (Figure 1a). The pump (*λ* ~800–950 nm, pulse duration ~10 fs) and probe pulses (*λ* ~700–750 nm, pulse duration ~20 fs) were further spectrally filtered using bandpass (BP) filters to avoid any spectral overlap and interference artifacts between them. Their respective spectra are shown in Figure 1b. The probe pulse was delayed using a precision motorized linear stage before being recombined with the pump pulse. The collinearly propagating pump and probe pulses were then focused onto the sample using a reflective objective (numerical aperture of 0.5). The light emanating from the sample was collected using the same objective and passed through a 690 nm shortpass filter (SP) before being guided into a spectrometer.

**FIGURE 1:**
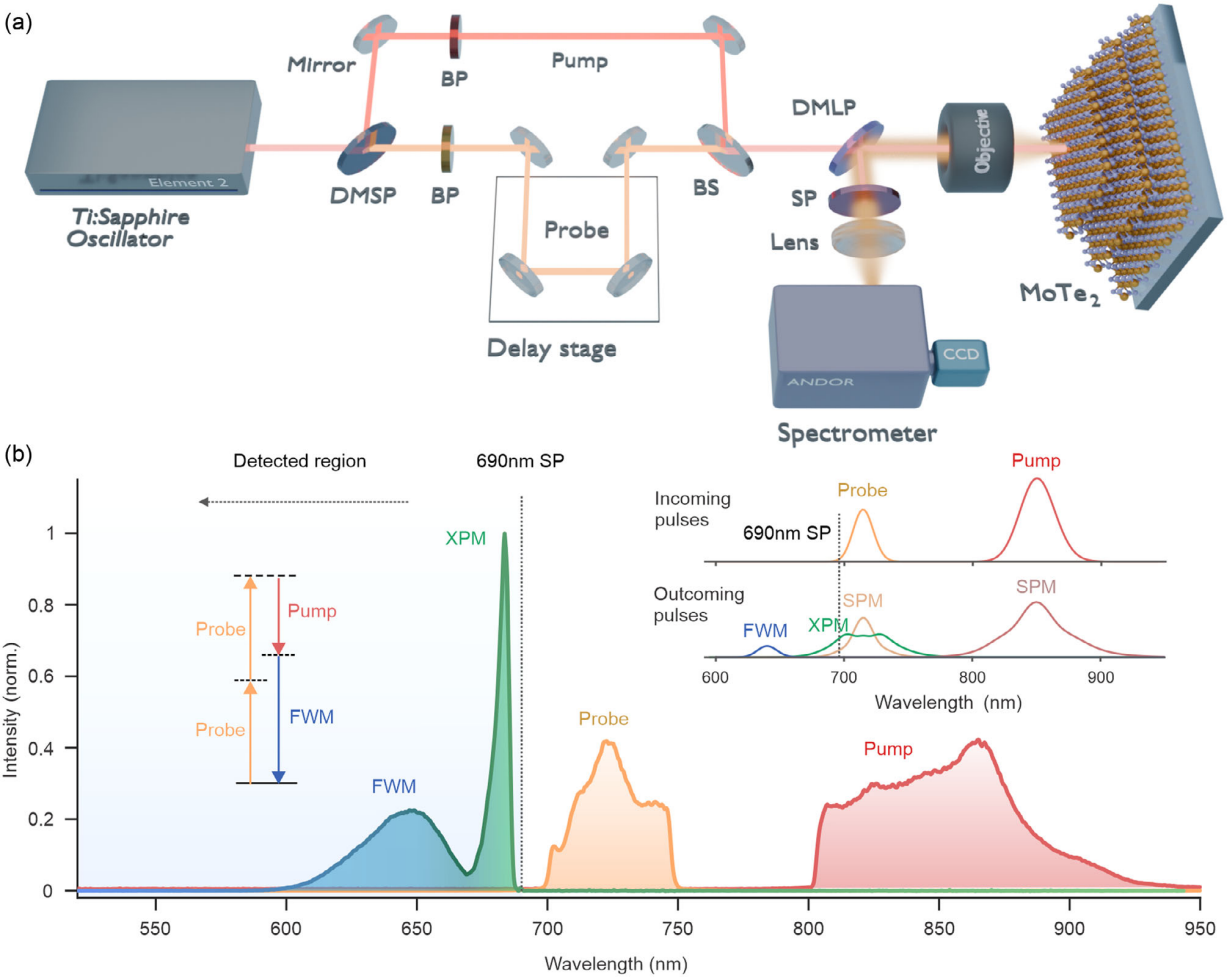
Phase‐sensitive nonlinear spectroscopy of ultrathin 2*H*‐MoTe_2_. (a) Schematic illustration of phase‐sensitive nonlinear pump‐probe spectroscopy in a chemical vapor deposition (CVD)‐grown few‐layer (three to five) thick 2*H*‐MoTe_2_ on a quartz substrate. Collinearly propagating nondegenerate pump (~800–950 nm, ~10 fs) and probe (~700–750 nm, ~20 fs) laser pulses were focused onto the sample using a reflective objective. The light emanating from the sample was collected using the same objective, filtered through a 690 nm shortpass filter to remove residual incident laser components, before being focused into the spectrometer. BP, bandpass filter; SP, shortpass filter; DMSP, shortpass dichroic mirror; DMLP, longpass dichroic mirror. (b) Spectra of the incoming pump (red filled curve) and the probe (orange filled curve) pulses, shown on the right side of the dashed black curve, together with the measured spectral broadening of the probe pulse induced by cross‐phase modulation (XPM, green filled curve) and the four‐wave mixing (FWM, blue filled curve) processes on the left side of the dashed black curve. Left inset: schematic of the FWM contribution to the measured spectra, resulting from interaction of two photons from the probe pulse (upward transitions) followed by interaction with one pump photon (downward transition). Right inset: spectral‐domain illustration of the self‐phase modulation (SPM), XPM, and the FWM processes. Incoming pump and probe pulses undergo SPM as well as XPM, resulting in broadened spectra. Spectral components present on the left side of the dashed black curve were measured in the experiments, comprising SPM of the probe pulse, XPM, and the FWM signals. SPM of the pump pulse was not measured as it lies outside the measured spectral window.

A probe pulse propagating through the 2*H*‐MoTe_2_ sample experiences a time‐varying refractive index owing to the Kerr effect [27], thereby inducing a time‐dependent phase shift across the pulse envelope [28]

(1) $$\begin{array}{c} \text{Δ}\phi (t)=\frac{2\pi }{\lambda }n_{2}I(t)L \end{array}$$

where λ is the wavelength of incident light, $n_{2}$ is the nonlinear refractive index coefficient of 2*H*‐MoTe_2_, I(t) is the instantaneous intensity of the probe pulse, and *L* is the effective interaction length within the material. This phase shift leads to spectral broadening (see Figure S1), characterized by the instantaneous frequency change

(2) $$\begin{array}{c} \delta \omega \left(t\right)=-\frac{\partial \text{Δ}\varphi \left(t\right)}{\partial t} \end{array}$$

When both the pump and probe pulses copropagate through the nonlinear medium, the phase shift experienced by the probe pulse is influenced by the combined intensity of both pulses. Consequently, the time‐dependent phase shift acquired by the probe pulse becomes

(3) $$\begin{array}{c} \text{Δ}\phi \left(t\right)=\frac{2\pi }{\lambda }n_{2}\left(I_{\text{probe}}\left(t\right)+\kappa I_{\mathrm{pump}}\left(t\right)\right)L \end{array}$$

where κ is a coefficient, often close to 2 in different materials [28, 29, 30], indicating that the pump pulse exerts a stronger influence on the probe pulse than the probe pulse does on itself (SPM). This interaction gives rise to an intense XPM signal at the zero‐time delay between the pulses, as shown by the green‐shaded spectral region in Figure 1b and the inset (right) sketch. Moreover, when the pump and probe pulses temporally overlap, four‐wave mixing (FWM) processes also occur. One prominent case involving two photons from the probe pulse and one photon from the pump pulse results in the blue‐shaded spectrum in Figure 1b, as depicted in the inset (left).

The nonlinear XPM and FWM responses were characterized by varying the delay between the pump and the probe pulses. Figure 2a shows a series of pump‐probe spectra, in which a spectrum measured at far negative delay of −1 ps between the pulses was subtracted from each individual spectrum, effectively removing the SPM signal generated by the probe pulse alone. Owing to the broad spectral separation between the pump and probe pulses, SPM induced solely by the pump pulse is not detected. At zero delay, pronounced nonlinear signals emerge, corresponding to XPM (~670–690 nm) and FWM (~620–670 nm). Following the initial pump‐probe overlap, the XPM signal exhibits a sharp drop in its amplitude followed by periodic oscillations lasting up to 10 ps. The initial decrease and recovery of the XPM signal at longer delays are attributed to an ultrafast electronic response, where depletion of the carriers in the valence band upon photoexcitation with the pump pulses reduces the Kerr nonlinearity [31].

**FIGURE 2:**
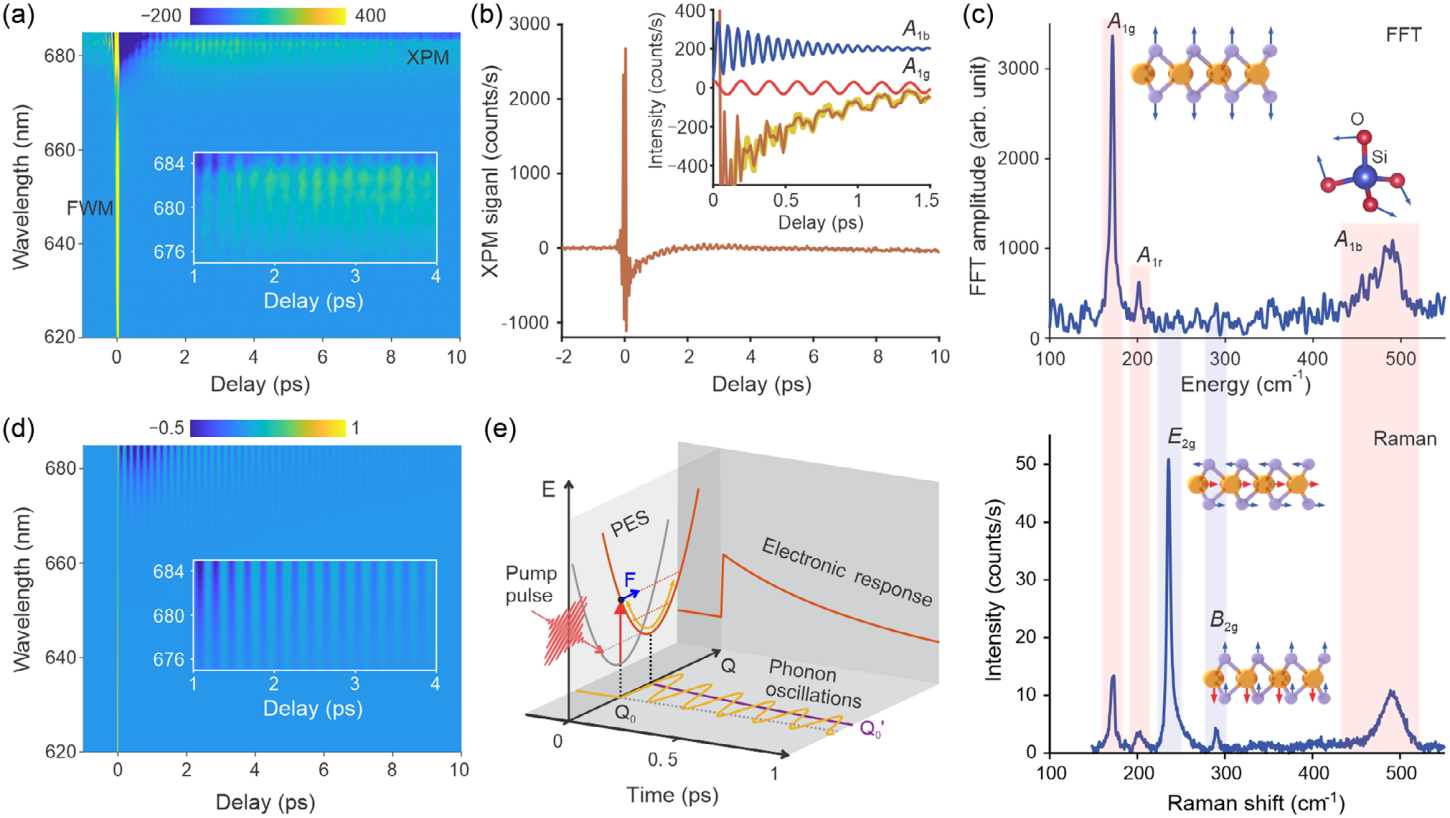
Ultrafast modulation of Kerr nonlinearity via coherent phonons. (a) A series of spectra measured as a function of the delay between the pump and the probe pulses. The spectrum recorded at −1 ps delay was subtracted from each spectrum, in order to remove the contribution of the SPM of the probe pulse, to generate a purely XPM spectral trace. SPM of the pump pulse was not measured as it lies relatively far from the edge of the measured spectral window. Inset shows the close‐up of the measurement after the zero‐time delay. (b) Temporal cross‐cut of the XPM spectra at ~680 nm from the measurement shown in (a). Pump and probe laser powers were set to 2.1 mW (26 pJ) and 3.0 mW (37.5 pJ), respectively. Inset: simulation (yellow) of the experimental time trace (orange) by summing up two damped oscillations, representing the *A*_1g_ (red) and *A*_1b_ (blue) phonon modes. (c) Top panel: fast Fourier transform (FFT) of the time trace in (b) in the delay range of 0.1–10 ps. The FFT spectrum reveals a strong peak at ~172 cm^−1^, corresponding to the *A*_1g_ phonon mode of 2*H*‐MoTe_2_, and a broad peak at ~480 cm^−1^, attributed to the *A*_1b_ phonon mode of the quartz substrate. Bottom panel: Raman spectrum of the 2*H*‐MoTe_2_ sample on quartz, measured with a 633 nm CW laser excitation source. A laser power of 0.89 mW was used in the measurement. (d) Simulated pump‐probe spectra incorporating both XPM and FWM contributions. Inset shows the close‐up of the simulation after the zero delay. (e) Schematic illustration of the displacive excitation of the coherent phonons. Upon excitation with the pump pulse (red), the potential energy surface (PES) of the system shifts within the Born–Oppenheimer approximation, resulting in a new equilibrium position (*Q*_0_′) of the nuclei. This sudden shift in the PES exerts a fully symmetrical force, *F*, on the lattice, initiating coherent phonon oscillations (yellow). The PES (orange) and equilibrium position of the nuclei (purple) evolve in time.

To analyze the oscillatory behavior in detail, the temporal crosscut of the pump‐probe XPM spectra at ~680 nm is plotted in Figure 2b. The time trace exhibits a strong peak at the zero‐time delay, followed by an abrupt reduction and a subsequent recovery within ~1 ps, superimposed with long‐lived oscillations. Fast Fourier transform (FFT) analysis of this time‐domain signal reveals a pronounced peak at ~172 cm^−1^ (upper panel in Figure 2c), corresponding to the out‐of‐plane *A*_1g_ phonon mode of 2*H*‐MoTe_2_. This mode exhibits a long coherence lifetime (~5.0 ps), consistent with previous transient reflection measurements [32]. In addition, our data also reveal a broad peak centered at ~480 cm^−1^, indicating a short‐lived phonon mode (~0.4 ps, see also the time–frequency analysis of the pump‐probe traces in Figure S2). This spectral peak is attributed to the *A*_1b_ mode of the quartz substrate, a ring O_bridge_‐breathing phonon mode [33].

Apart from these dominant phonon modes, we also observed a weak spectral peak at ~202 cm^−1^ (Figure 2c) that matches the *A*_1r_ (rotation mode of SiO_4_ tetrahedra) mode of quartz [33]. These mode attributions were further confirmed by Raman spectroscopic characterization performed on the same sample (lower panel in Figure 2c). Notably, few‐layer 2*H*‐MoTe_2_ transferred onto a Si_3_N_4_ substrate shows neither the *A*_1b_ nor the *A*_1r_ mode (see Figure S3). The presence of the phonon modes of the quartz substrate in the time‐resolved measurements corroborates the exceptional phase sensitivity of the present XPM‐based technique.

A cosine‐like oscillation can be assigned to both the *A*_1g_ mode of 2*H*‐MoTe_2_ and *A*_1b_ mode of quartz by fitting the time‐domain signal backwards to the zero delay with phases of (0.02±0.07)π, and (1.02±0.05)π, respectively, as shown in the inset of Figure 2b. The absolute maximum of the FWM signal in the measurements enables clocking of the zero‐time delay between the pump and probe pulses. The opposite phases at the zero‐time delay of the two phonon modes likely result from their distinct eigenvector orientations and differences in the underlying band structures [33, 34]. Notably, the strongest Raman‐active *E*_2g_ mode of 2*H*‐MoTe_2_ at ~240 cm^−1^ is absent in the time‐resolved XPM measurements (Figure 2c). A similar behavior was observed in few‐layer 1*T*‐MoTe_2_, where the *A*_g_ mode at ~253 cm^−1^ was observed without the presence of the corresponding *E*_2g_ mode (see Figure S3). Coherent phonon oscillations corresponding to the *A*_1_ mode were also measured for a monolayer 2*H*‐MoTe_2_ mechanically exfoliated onto a z‐cut sapphire substrate (see Figure S4). The selective mode excitation of fully symmetric *A* modes and their cosine‐like oscillatory behavior suggest a displacive excitation mechanism of phonons in MoTe_2_ and the underlying quartz substrate [35], as schematically shown in Figure 2e. Illumination with ultrafast laser pulses induces an instantaneous redistribution of electronic charge in *2H*‐MoTe_2_. This rapid electronic redistribution alters the potential energy surface (PES), *U*, on attosecond timescales [33], causing a shift in the equilibrium position (*Q*_0_) of the lattice within the adiabatic Born–Oppenheimer approximation. As a result, atoms are impulsively displaced from their original equilibrium positions and subsequently oscillate coherently around the new equilibrium position (*Q*_0_′) defined by the transient electronic state [34].

The oscillations in the XPM signal at the phonon frequencies of *2H*‐MoTe_2_ and quartz indicate that the pump pulses excite coherent phonons, which are subsequently detected by the probe pulse via phase‐sensitive nonlinear interactions. This interpretation is further attested by time‐dependent density functional theory (TDDFT) calculations; see Figure S5 and the Supporting Information for details. Upon interacting with the coherently oscillating lattice, the probe pulse acquires a periodic phase modulation, leading to a periodic shift in the COM of its spectral weight. This spectral shift manifests experimentally as oscillations in the broadened spectral intensity of the probe pulse [12]. By incorporating both FWM and XPM contributions into the simulations, the key features in the pump‐probe spectra can be reasonably reproduced, as shown in Figure 2d. The transient modulation of the Kerr nonlinearity by the pump pulse can be attributed to photoexcitation of the carriers and the resulting modifications of the electronic band structure [9, 31], which will be discussed later in the text. In the simulation shown in Figure 2d, the ultrafast electronic depletion is modeled using a step‐like response followed by an exponential decay [9, 31, 35]; see Methods for details.

The presence of extensive dynamical features in the pump‐probe traces demonstrates that the probe pulse is highly sensitive to pump‐induced modifications of the material dielectric function [36]. Compared with other nonlinear techniques, such as high‐harmonic generation [33, 37], transient reflection, transmission, and absorption spectroscopy [38], this phase‐sensitive approach enables coherent phonon detection with pulse energies as low as a few picojoules and a strongly suppressed background while providing high sensitivity to pump‐induced modulation (see Table S1 for details). The use of low pulse energies helps minimize thermal degradation and sample damage, which are particularly relevant for low‐dimensional materials and devices. Optical absorption and inefficient heat dissipation can produce substantial temperature‐dependent changes in the optical properties. Although such photothermal effects can be exploited for all‐optical tuning and related photonic functionalities [21], excessive optical heating may lead to degradation or damage of the material and device performance. In the present study, the use of picojoule pulse energies keeps the average optical power in the milliwatt range thereby helping to reduce cumulative heating while retaining sensitivity to ultrafast nonlinear optical response. The phase‐sensitive XPM approach therefore offers a useful tool for resolving quasiparticle interactions, exciton formation, and their relaxation dynamics in quantum materials.

## 3 Displacive Excitation of Symmetric Phonon Modes

To elucidate the mechanism underlying the coherent phonon excitation, we performed fluence‐dependent measurements. First, we varied the fluence of the probe pulse while keeping the pump fluence fixed. The spectral intensities of the retrieved phonon modes, *A*_1g_ and *A*_1b_, exhibit a quadratic dependence on the probe fluence, as shown in Figure 3a. Similarly, the amplitude of the depleted XPM signal at the zero pump‐probe delay also exhibits a quadratic dependence on the probe fluence (blue curve, Figure 3b). The retrieved quadratic scaling of the intensity of the phonon modes rules out the FWM (or coherent anti‐Stokes Raman (CARS)) process involving two photons from the pump pulse and one photon from the probe pulse as the origin of the vibrational coherence (see also Figure S6) [39, 40]. It is worth mentioning that the anti‐Stokes Raman signal would be fully suppressed in our experimental configuration (see Supporting Information for details). The observed FWM process at ~650 nm, involving two photons from the probe pulse and one photon from the pump pulse, exhibits the expected quadratic scaling on probe fluence (see Figure S7). The nonlinear dependence of both the XPM response and phonon amplitudes on probe fluence strongly supports the nonlinear XPM mechanism. It is worth noting that the decay constants associated with the relaxation times of the photocarriers exhibit a decreasing trend on increasing probe fluence (red curve in Figure 3b), reflecting the role of enhanced carrier–carrier interactions at higher photoexcitation intensities on the retrieved relaxation times.

**FIGURE 3:**
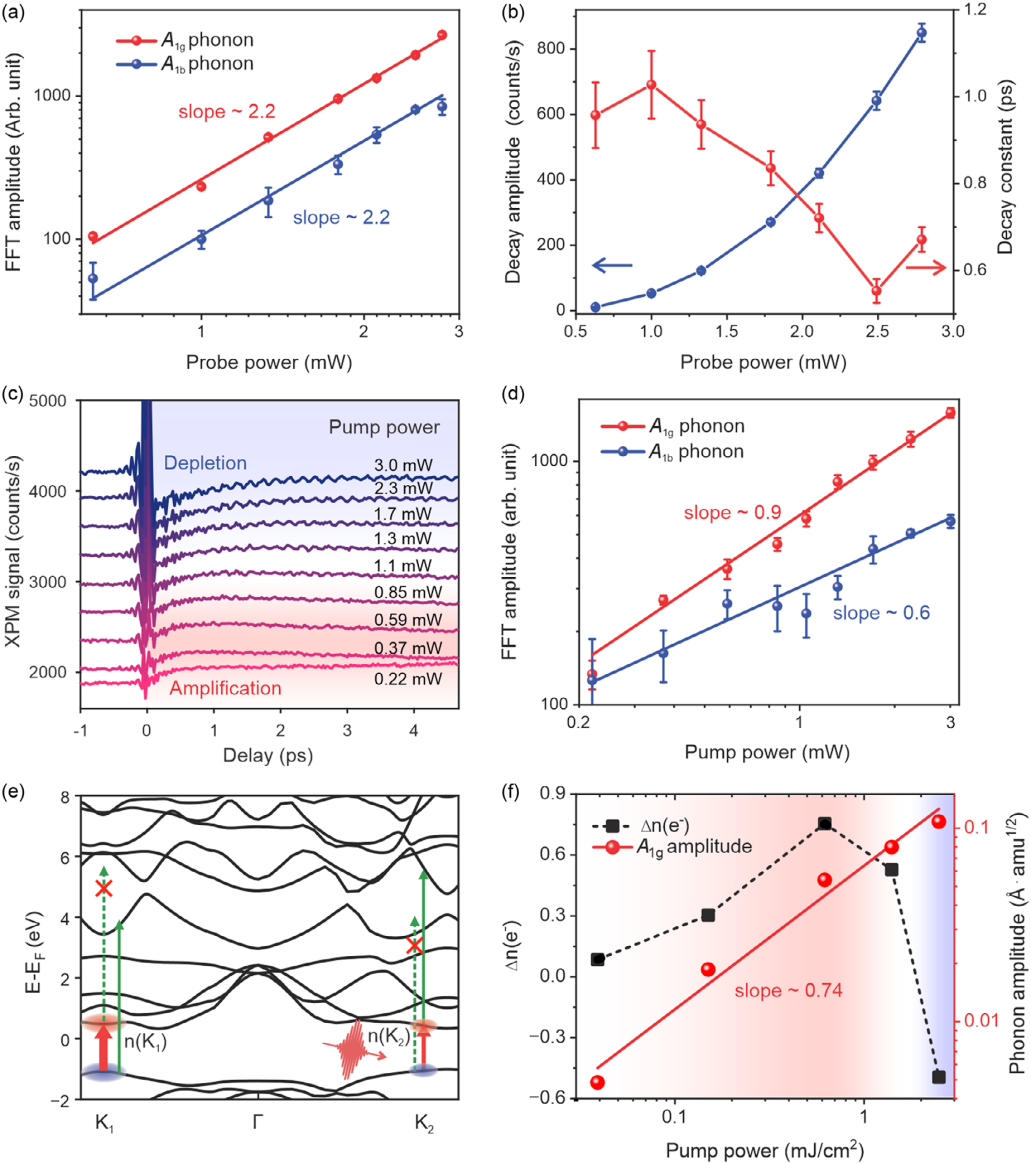
Evolution of Kerr nonlinearity with the fluence of the driving pulses and displacive excitation of coherent phonons. (a) Variation of the spectral intensities of the *A*_1g_ (red dots) and *A*_1b_ (blue dots) phonon modes on increasing the fluence of the probe pulse. Phonon amplitudes were obtained from the FFT analysis of the time‐resolved pump‐probe traces (see Figure S8). Red and blue curves represent fits to the data in the dual‐logarithmic plots, with fit parameters annotated in the respective legends. The power of the pump pulse was kept at 2.2 mW, and an acquisition time of 0.5 s per delay step was used for all spectra. (b) Variation of the amplitudes of the XPM signal at the zero delay between the pump and probe pulses (blue dots), and the decay time constants (red dots) on increasing the fluence of the probe pulse, extracted from the time traces shown in Figure S8. (c) A series of time‐resolved XPM measurements acquired at increasing pump fluence; the XPM signal was enhanced (red area) at low pump fluence but became depleted (blue area) at high pump fluence. The fluence of the probe pulse was fixed at 2.1 mW. Pump fluences are annotated on each time trace, which are vertically shifted for clarity. (d) Variation of the amplitudes of the *A*_1g_ (red dots) and *A*_1b_ (blue dots) phonon modes as a function of pump fluence, as retrieved from the FFT analysis of the measurements shown in (c). Red and blue curves display the nonlinear fits in the dual‐logarithmic plots, with the fit parameters displayed in the respective legends. (e) Density functional theory (DFT)‐calculated band structure of *2H‐*MoTe_2_. Under pump pulse excitation, electrons at the *K*_1_ and *K*_2_ points are resonantly promoted to the conduction band (red arrows). The contribution of the probe pulse to XPM arises primarily from resonant interband transitions (solid green arrows). (f) TDDFT‐calculated evolution of the population difference of photoexcited electrons at the *K*_1_ and *K*_2_ points, $\text{Δ}n=n_{K2}-n_{K1}$ (black squares, left *y*‐axis), as a function of pump fluence. The evaluated *A*_1g_ phonon amplitudes (red dots, right *y*‐axis) are plotted over the same pump‐fluence range. The double‐logarithmic fit (solid red curve) of the evaluated phonon amplitudes yields a slope of 0.74, indicating an approximately linear dependence on the pump fluence.

The excitation of coherent phonons upon interaction with ultrashort laser pulses typically arises from two plausible mechanisms: (1) impulsive stimulated Raman scattering (ISRS) or (2) displacive excitation of coherent phonons (DECP) [32]. To further distinguish between them, we performed time‐resolved XPM measurements by varying the pump fluence, while keeping the fluence of the probe pulse fixed. The temporal crosscuts at ~680 nm from the pump‐probe spectra are shown in Figure 3c. Compared to the probe‐fluence‐dependence measurement, the time‐resolved traces show markedly more complex behavior (see time traces in Figure S8). At low fluence of the pump pulse, the XPM signal gradually increases after the zero‐time delay (bottom curves in Figure 3c), in contrast with the abrupt reduction observed in Figure 2a,b. As the pump fluence increases, this gradual rise in the XPM signal, after the zero‐time delay, transitions into a pronounced depletion (top curves in Figure 3c). The evolution from amplification to attenuation of the XPM signal reflects a pump‐induced modification of the complex nonlinear optical response. While the spectral redistribution and shift of the COM are characteristic of the nonlinear phase modulation, associated predominantly with the real part of *χ* ^(3)^, absorptive contributions associated with the imaginary part of *χ* ^(3)^ and the pump‐induced changes in the complex dielectric function may also contribute to the measured spectral intensity [22]. While this behavior complicates the extraction of the decay constants of the electronic response, phonon amplitudes can still be robustly quantified via the FFT analysis of the time‐domain traces. The extracted amplitudes of both the *A*_1g_ and *A*_1b_ modes exhibit an approximately sublinear dependence on the pump fluence (Figure 3d), which is consistent with the DECP mechanism [35].

Within the DECP framework, the uniform photoexcitation of electrons creates a symmetric driving force: $F\left(t\right)=-\frac{\partial U(t)}{\partial Q}$, leading to the selective excitation of fully symmetric vibrational modes, such as the *A*_1g_ mode in MoTe_2_, whose atomic displacements match the symmetry of the perturbation. In contrast, low‐symmetry modes, such as doubly degenerate *E*‐type modes, which involve antisymmetric or degenerate atomic motions, are not efficiently excited, as their displacements do not align with the symmetry of the driving force [41]. This symmetry‐selective excitation mechanism explains the exclusive observation of fully symmetric *A* modes in both 2*H*‐MoTe_2_ and quartz (a similar trend is also observed in 1*T*‐MoTe_2_, see Supporting Information for details).

## 4 Amplification and Attenuation of the Kerr Nonlinear Response

We now focus on the amplification and attenuation behavior of the Kerr nonlinear response induced by the nonlinear interaction of the probe pulse following excitation by the pump pulse, as highlighted by the red and blue areas in Figure 3c. The interband contribution to the imaginary part of the third‐order nonlinear susceptibility, $\chi_{\text{inter}}^{\left(3\right)}$, can be expressed in terms of the electronic distribution in different bands as [42]

(4) Imχinter(3)∝∑i,j∫d3k4π3|Pij(k)|4×fi(k)(1−fj(k))×δ(Ej(k)−Ei(k)−ℏω)

where $P_{ij}$ denotes the matrix element of the momentum operator, and $f_{i}$ and $f_{j}$ are the occupation probabilities of the initial (*i*) state and final (*j*) states. Thus, photoexcitation directly modifies the nonlinear refractive index *n*_2_ via changes in the electronic population. In the present case, we focus on the *K*_1_ and *K*_2_ points in the band structure, where the pump pulse (~1.3–1.55 eV) resonantly excites electrons from the valence band to the conduction band, as indicated by the vertical red arrows in Figure 3e.

In this case, the pump‐induced modification in Kerr nonlinearity mainly arises from electronic populations at the *K*_1_ and *K*_2_ points. At the *K*_1_ point, the valence band states deplete under resonant pump excitation (blue area on the left side of Figure 3e); nonetheless, the remaining electrons at the *K*_1_ point can still participate in the XPM process via resonant interband transitions (solid green arrow on the left side of Figure 3e). However, this transition probability would be reduced on increasing the pump fluence, because a fraction of valence‐band electrons would have already been promoted to the conduction band. This results in a reduction in the *χ* ^(3)^ contribution and leads to a suppression of the nonlinear signal, consistent with the reduced XPM response observed in Figure 2a,b.

Nevertheless, photoexcited electrons in the conduction band, unoccupied in equilibrium, can amplify the nonlinear response. This amplification occurs predominantly at the *K*_2_ point, where pump‐excited carriers (red area on the right side of Figure 3e) enable resonant probe‐induced *χ* ^(3)^ processes involving higher conduction band states (solid green arrow on the right side of Figure 3e). This contribution effectively increases the Kerr nonlinearity and the associated XPM signal.

The interplay between the above two processes gives rise to the nonmonotonic fluence dependence recorded in Figure 3c. Upon weak pump pulse excitation, photoexcited carriers at the *K*_2_ point ($n_{K2}$) enhance the nonlinear response experienced by the probe pulse, whereas the carrier population in the conduction band near *K*_1_ remains largely unchanged. On increasing the pump fluence, resonant depletion of valence band states near *K*_1_ point $\left(n_{K1}\right)$ dominates, leading to a net reduction of the XPM signal. The carrier population imbalance,$\;\text{Δ}n=n_{K2}-n_{K1}$, as captured by the TDDFT calculations, is shown by the black squares in Figure 3f. It increases at low pump fluence (red area) and decreases at higher fluence (blue area), following the nonmonotonic evolution of the measured XPM response. This correlation demonstrates that the phase‐sensitive XPM approach is also sensitive to the transient electronic contribution to the nonlinear optical response.

In contrast to the complex nonlinear electronic response, we find that the phonon amplitude increases monotonically with pump fluence, as shown by the red dots in Figure 3f, in agreement with the measured phonon amplitudes shown in Figure 3d. These results suggest that electronic and lattice contributions to the nonlinear optical response exhibit distinct pump‐fluence dependences and can therefore be separately resolved, and their responses can be sensitively controlled using even a weak optical gating pulse [33, 35, 41]. This capability is highly relevant to the broader development of ultrafast nonlinear and integrated photonics in low‐dimensional materials.

## 5 Coherent Control of Phonon Oscillations via a Dual‐Pump Scheme

Having established the excitation and detection mechanisms of the coherent phonons, we next examine their controlled manipulation [43, 44, 45, 46], which is crucial in shaping the physical properties of materials on terahertz timescales. Controlled phonon dynamics is particularly relevant in phenomena such as charge density wave formation, excitonic states [47, 48, 49], and electron pairing in superconductors [48]. To achieve this, we employ two identical pump pulses, hereafter referred to as “pump‐1” and “pump‐2,” each capable of individually exciting coherent phonons. By precisely tuning the delay between these two pump pulses, phonon oscillations can be selectively enhanced or suppressed through constructive or destructive interference of the launched phonon wavepackets, as illustrated in Figure 4a. The phase‐sensitive modulation of the probe pulse enables real‐time monitoring of this interference of the phonon wavepackets.

**FIGURE 4:**
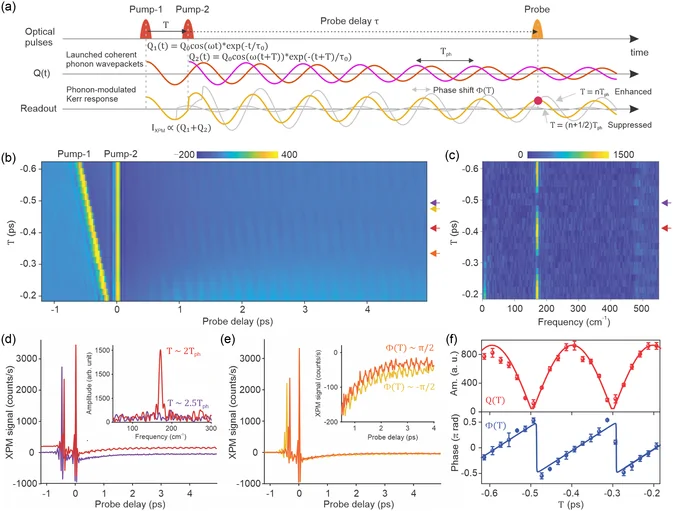
All‐optical coherent control of Kerr nonlinearity via a dual‐pump pulse scheme. (a) Schematic illustration of coherent control of phonon dynamics using two pump pulses (“pump‐1” and “pump‐2”), which launch coherent phonon wavepackets $Q_{1}(t)$ (red) and $Q_{2}(t)$ (magenta), respectively. The relative delay *T* between the two pump pulses controls the phase (Φ(T)) and amplitude (Q(T)) of the launched phonons and, in turn, modulates the phonon‐driven Kerr nonlinearity of 2*H*‐MoTe_2_ probed at a later delay *τ* by the probe pulse. When $\text{T}=nT_{\text{ph}}$, the phonon response is enhanced; when $T=\left(n+\frac{1}{2}\right)T_{\mathrm{ph}}$, it is suppressed, where $T_{\mathrm{ph}}$ is the phonon oscillation period. (b) A series of time‐resolved traces measured as a function of the delay between the pump‐1 (and pump‐2) and probe pulses for various delays between the pump‐1 and pump‐2 pulses (*T*), as annotated along the *y*‐axis. The temporal crosscuts were extracted at ~680 nm from the dual‐pump‐probe measurements. The pump‐1 and pump‐2 pulse powers were set at 3.4 mW (43 pJ), and the probe pulse power was set at 2.0 mW (25 pJ). The acquisition time was 0.5 s per delay step for all spectra. (c) FFT spectra of the time‐resolved traces in (b). (d) Comparison of the time‐resolved traces measured at two fixed delays between pump‐1 and pump‐2 pulses (*T* = −0.42 and −0.49 ps) as indicated by the red and purple arrows in (b), demonstrating the intensity control of the *A*_1g_ phonon mode by varying the delay between the two pump pulses. Inset: FFT spectra of the two time‐resolved traces show phonon oscillations being completely activated ($T\sim \;2T_{\mathrm{ph}}$) or suppressed ($T\sim \;2.5T_{\mathrm{ph}}$). (e) Comparison of the time‐resolved traces measured at two fixed delays between the pump‐1 and pump‐2 (*T* = −0.31 and −0.47 ps) pulses as indicated by the orange and yellow arrows in (b), demonstrating phase control of the *A*_1g_ phonon mode by adjusting the delay between the two pump pulses. Inset: A close‐up of the phonon oscillations, highlighting the out‐of‐phase oscillation behavior. (f) The amplitude (Q(T), upper panel, red dots) and phase (Φ(T), lower panel, blue dots) of the *A*_1g_ phonon mode in 2*H*‐MoTe_2_ as retrieved from the measurement shown in (b) as a function of T. The solid red and blue curves represent simulations based on two damped harmonic oscillators driven by a pair of pump pulses.

Figure 4b demonstrates phonon control by varying the delay, T, between the pump‐1 and pump‐2 pulses. In each time trace, the delay between these two pump pulses was fixed as labeled along the vertical *y*‐axis, whereas the delay between the two pump pulses and the probe pulse was varied along the horizontal *x*‐axis. The individual time traces in Figure 4b represent temporal crosscuts at 680 nm from the pump‐probe measurements. The amplitudes of the phonon oscillations exhibit a clear periodic modulation as a function of T. The FFT spectra (Figure 4c) of these time traces reveal that the amplitude of the *A*_1g_ mode oscillates with a period of $T_{\mathrm{ph}}$ ~194 fs, corresponding to the *A*_1g_ phonon mode frequency (172 cm^−1^). At specific pump–pump delays, the oscillations are largely enhanced ($\;T\;\sim \;2T_{\mathrm{ph}}$, red arrows in Figure 4b), whereas at other delays, they are almost completely suppressed ($\;T\;\sim \;2.5T_{\mathrm{ph}}$, purple arrows in Figure 4b). The representative time traces and their FFT spectra (inset) are shown in Figure 4d.

In addition to the amplitude control, the phase of the phonon oscillations can also be tuned by adjusting the delay between the pump‐1 and pump‐2 pulses, allowing the oscillations to be driven completely in or out of phase, as shown in Figure 4e. This behavior is extracted from the time traces indicated by the orange and yellow arrows in Figure 4b. The interference of the phonon wavepackets leading to variation in the amplitude (red dots) and phase (blue dots) of the *A*_1g_ phonon mode can be modeled using two driven damped harmonic oscillators [50, 51]

(5) $$\begin{array}{c} Q_{\upsilon }\left(t,T\right)=Q_{\upsilon ,0}\cos\;\left(\omega_{\upsilon }t\right)e^{-\frac{t}{\tau_{p}}}+Q_{\upsilon ,0}\cos\;\left({\text{ω}}_{\upsilon }(t-T\right))e^{-\frac{\left(t-T\right)}{\tau_{p}}} \end{array}$$

where $Q_{\upsilon ,0}$ is the initial amplitude set by DECP excitation, ${\text{ω}}_{\upsilon }$ is the phonon oscillation frequency, and $\tau_{p}$ is the phonon decay time constant. The resulting phase of the phonon oscillation is given by

(6) $$\begin{array}{c} {\text{Φ}}_{\upsilon }\left(T\right)=-\arctan\;\left(\frac{\sin\;\left({\text{ω}}_{\upsilon }T\right)e^{\frac{T}{\tau_{p}}}}{\cos\;\left({\text{ω}}_{\upsilon }T\right)e^{\frac{T}{\tau_{p}}}+1}\right)\; \end{array}$$

Using this model, we accurately reproduce the experimentally observed amplitude and phase modulation, as shown by the solid curves in Figure 4f. The experimentally observed phonon control is also independently confirmed by TDDFT calculations (see Supporting Information for details).

This phonon control offers a novel approach for manipulating dynamics in materials. By selectively turning phonon oscillations on or off, the electronic response can also be modulated, as evidenced by the gain in the XPM signal observed at shorter delays between the pump‐1 and pump‐2 pulses in Figure 4b. The relatively slow modulation of the Kerr nonlinear response leads to the emergence of a very low‐frequency mode in the FFT spectra in Figure 4c. Such precise control over phonons holds promise for manipulating nonequilibrium phases in MoTe_2_, such as phonon‐mediated superconductivity and phase transitions [25, 52].

## 6 Concluding Remarks

In this work, we demonstrated a phase‐sensitive nonlinear optical spectroscopic technique capable of providing direct insights into ultrafast electronic and phononic dynamics. We showed that optically excited coherent phonons modulate the Kerr nonlinearity of materials, inducing cross‐phase modulation on a copropagating probe pulse. This approach operates with minimal pulse power is applicable to various materials and could provide a sensitive probe of transient phase transitions and excitonic dynamics in a nondestructive manner. Coherent control of phonon oscillations was achieved using a dual‐pump pulse scheme, enabling precise real‐time manipulation of phonon dynamics. This capability lays the foundation for further exploration of phonon‐mediated control in quantum materials, such as charge density waves and superconductivity. Our findings open new avenues for phonon‐engineered light‐matter interactions on femtosecond timescales and highlight the potential of ultrafast nonlinear optical modulations for advancing next‐generation quantum and optoelectronic devices.

## 7 Methods

### 7.1 Experimental Setup

Ultrafast pump (*λ* ~800–950 nm, pulse duration ~10 fs) and probe pulses (*λ* ~700–750 nm, pulse duration ~20 fs) were employed for the phase‐sensitive nonlinear spectroscopy measurements. The pump and probe pulses were spectrally separated from the ultrabroadband output of a Ti:sapphire oscillator (Element 2) using an 800 nm shortpass dichroic mirror (DMSP) and were precompressed with a pair of chirped mirrors. The laser was operated at a repetition rate of 80 MHz for all measurements. For a pulse energy of ~10 pJ and a focal spot diameter of ~3–4 μm at the sample, the estimated pulse fluence was ~100 μJ/cm^2^.

The collinearly propagating pump and probe pulses were focused onto the sample (mounted onto a motorized 3D stage) using a 40 × reflective objective with a numerical aperture of 0.5. The light emanating from the sample was then collected using the same objective and passed through a 690 nm shortpass filter (SP) before being directed into a spectrometer for analysis. A precision motorized linear stage (PI Instruments) was used to adjust the delay between the pump and probe pulses. For all spectra, an acquisition time of 0.5 s per delay step was used unless otherwise specified.

For pump‐probe experiments, the power of both pump and probe pulses could be independently controlled using two motorized neutral density (ND) filters in their respective beam paths. Raman spectroscopy was performed on a commercial Raman spectrometer from WiTec, and a 633 nm CW laser was used for the measurements. The laser power was kept below 1 mW to avoid sample damage. All the experiments were conducted at room temperature.

### 7.2 Sample Preparation

The few‐layer (three to five layers thick) 2*H*‐MoTe_2_ samples, grown by chemical vapor deposition (CVD) on a quartz substrate, were purchased from 2D Semiconductors. The 2*H*‐MoTe_2_ and 1*T*‐MoTe_2_ flakes (in Figure S3) were mechanically exfoliated from their bulk crystals (bought from HQ Graphene) and transferred onto the Si_3_N_4_ substrate within a glove box. The monolayer 2*H*‐MoTe_2_ was mechanically exfoliated and transferred onto a sapphire substrate, and its thickness was characterized using Raman spectroscopy (as shown in Figure S4). We note that no irreversible changes in the optical response or sample damage was observed under the experimental conditions used in this study.

### 7.3 Pump‐Probe Spectral Simulation

In the pump‐probe experiments, nonlinear cross‐phase modulation (XPM) and four‐wave mixing (FWM) processes occur as the pump and probe pulses interact in the nonlinear medium. For the XPM spectra simulation in Figure 2d, the Kerr nonlinearity response was separated into phonon and electronic contributions [35]. The phase response modulated by phonon oscillations was modeled as a damped oscillator

$$\Phi_{p,\nu }\left(t,\tau \right)=a\cdot \cos\;\left(\omega_{\nu }\left(t+\tau \right)+\phi_{v}\right)e^{-\frac{t+\tau }{\tau_{p}}}$$

where *a* is a prefactor, ${\text{ω}}_{\nu }$ is the phonon oscillation frequency, $\phi_{\nu }$ is the phase of the phonon oscillation, and $\tau_{p}$ is the phonon decay time constant determined from the phonon decay analysis in Figure S2.

The phase response contributed by electronic components was modeled as a simple step‐decay exponential function upon the pump pulse excitation

$$\Phi_{e}\left(t,\tau \right)=b\cdot u\left(\tau \right)e^{-\frac{t+\tau }{\tau_{e}}}$$

where *b* is a prefactor, u(τ) (u(τ≤0)=0,u(τ>0)=1) is a step function, and $\tau_{e}$ is the electronic response decay time constant determined from the exponential fit in Figure 2b. In the simulation, *a* and *b* are the only free fit parameters, which are related to the pump laser power.

FWM involves the interaction of the fields with nonlinear polarization of the medium, which results in the generation of new optical fields. In the simulations, the FWM processes that could generate photon energies larger than 1.77 eV (*λ* < 700 nm) were considered, as shown in Figure S6. The nonlinear polarization in the medium responsible for the FWM processes could be expressed in terms of the third‐order susceptibility $\chi^{\left(3\right)}$, which is given by

$$P=\sum_{j,k,l}\varepsilon_{0}\chi_{ijkl}^{\left(3\right)}E_{j}E_{k}E_{l}$$

where *i*, *j*, *k*, and *l* represent the Cartesian components of the nonlinear polarization and electric fields, respectively. The generated field at $\omega_{i}=\pm \omega_{j}\pm \omega_{k}\pm \omega_{l}$ is determined by the pump and probe field frequencies.

By adjusting the free parameters *a* and *b* in the XPM simulation, both the FWM and phonon oscillations in Figure 2a could be reproduced by the model described above.

### 7.4 Time‐Dependent Density Functional Theory (TDDFT)

The excited state dynamics can be simulated via a real‐time time‐dependent density functional theory framework (rt‐TDDFT), which we implemented in the QUANTUM ESPRESSO package [53, 54, 55].

Under the theorem of TDDFT, the time evolution of electron wave functions is governed by the time‐dependent Kohn–Sham (TDKS) equations [56]

$$i\frac{\partial }{\partial t}{\text{ψ}}_{i,k}\left(r,t\right)=\left[\frac{1}{2m}{\left(p-\frac{e}{c}A\right)}^{2}+V\left(r,t\right)\right]{\text{ψ}}_{i,k}\left(r,t\right),$$

where the velocity gauge is used and the external field appears in the kinetic term in the form of a vector potential **A**(*t*). The propagation of TDKS orbitals is implemented on the adiabatic basis $\phi_{n,k}(r,t)$

$$|{\text{ψ}}_{i,k}\left(r,t\right)\rangle =\sum_{n}c_{i,n,k}\left(t\right)|\phi_{n,k}\left(r,t\right)\rangle ,$$

where *i* and *n* denote the band indices, **k** refers to the reciprocal momentum index, and $c_{i,n,k}(t)$ are the time‐dependent coefficients. The adiabatic basis is calculated on the fly at each ionic step by diagonalizing the Hamiltonian

$$H_{k}\left(r,t\right)\left|\phi_{n,k}\left(r,t\right)\right\rangle ={\text{ε}}_{n,k}\left(t\right)\left|\phi_{n,k}\left(r,t\right)\right\rangle$$

where $\varepsilon_{n,k}(t)$ is the eigenvalue. The charge density can be calculated with $c_{i,n,k}(t)$ and $\phi_{n,k}(r,t)$

$$\text{ρ}\left(r,t\right)=\sum_{k}\sum_{n}q_{n,k}\left(t\right){\left|\left\langle \phi_{n,k}\left(r,t\right)|\phi_{n,k}\left(r,t\right)\right\rangle \right|}^{2},$$

where

$$q_{n,k}\left(t\right)=\sum_{i}|c_{i,n,k}\left(t\right){|}^{2},$$

is the population on the adiabatic basis.

For ions that are much heavier than electrons, their motions are treated classically on an averaged potential energy surface determined by the electronic distribution according to the Ehrenfest theorem. The nuclear positions are updated following Newton's second law [57]

$$M_{\text{α}}\frac{d^{2}R_{\text{α}}}{{\mathrm{dt}}^{2}}=\sum_{i}\left\langle \psi_{i}|\nabla_{R_{\alpha }}\left(\frac{1}{2m}{\left(p-\frac{e}{c}A\right)}^{2}+V\left(r,t\right)\right)|\psi_{i}\right\rangle$$

where $M_{\alpha }$ and $R_{\alpha }$ are the mass and position of the αth ion.

## Author Contributions

Shaoxiang Sheng, Yang Luo, Sayooj Sateesh, Marko Burkhard, Ashish Arora, and Manish Garg built the experimental setup, performed the experiments, and analyzed the experimental data. Chenyu Wang, Yaxian Wang, and Sheng Meng designed and performed the theoretical calculations and analyzed the theoretical data. Manish Garg conceived the project and designed the experiments. All authors interpreted the results and contributed to the preparation of the manuscript.

## Funding

S.M. acknowledges financial support from the National Natural Science Foundation of China (No. 12450401) and the Ministry of Science and Technology of China (No. 2021YFA1400201).

## Conflicts of Interest

The authors declare no conflicts of interest.

## Code Availability Statement

The details needed to reproduce the computations have been provided in the “Methods” section and Supplementary Information file.

## Supporting information

Supplementary Material

## Data Availability

The data that support the findings of this study are available from the corresponding authors upon request.
