# Supplementary material for: Phonon‐Modulated Kerr Nonlinearity in Ultrathin 2H‐MoTe2
Source: Small Sci. 2026 Oct 14;6(10):e70424. doi: 10.1002/smsc.70424 (PMC13669667; doi:10.1002/smsc.70424)
Supplement: Supplementary file 1 [file SMSC-6-e70424-s001.pdf]

Supplementary Information for

Phonon-modulated Kerr nonlinearity in ultrathin 2*H*-$MoTe_2$

Shaoxiang Sheng[1, 2]†*, Yang Luo[1, 3]†, Chenyu Wang[4,5]†, Sayooj Sateesh[1], Yaxian Wang[4], Marko Burkhard[1], Sayantan Patra[6], Bhumika Chauhan[6], Ashish Arora[6], Sheng Meng[4,5]*, Manish Garg[1,]*

[1]Max Planck Institute for Solid State Research, Heisenbergstr. 1, 70569 Stuttgart, Germany

[2]Tsientang Institute for Advanced Study, Hangzhou, 310024, China

[3]Hefei National Laboratory, University of Science and Technology of China, Hefei, 230088, China

[4]Beijing National Laboratory for Condensed Matter Physics and Institute of Physics, Chinese Academy of Sciences, Beijing 100190, China

[5]School of Physical Sciences, University of Chinese Academy of Sciences, Beijing 100190, China

[6]Indian Institute of Science Education and Research, Pune, 411008, India

*Corresponding authors. Email: s.sheng@fkf.mpg.de, smeng@iphy.ac.cn, mgarg@fkf.mpg.de

†These authors contributed equally to this work: S. Sheng, Y. Luo, and C. Wang

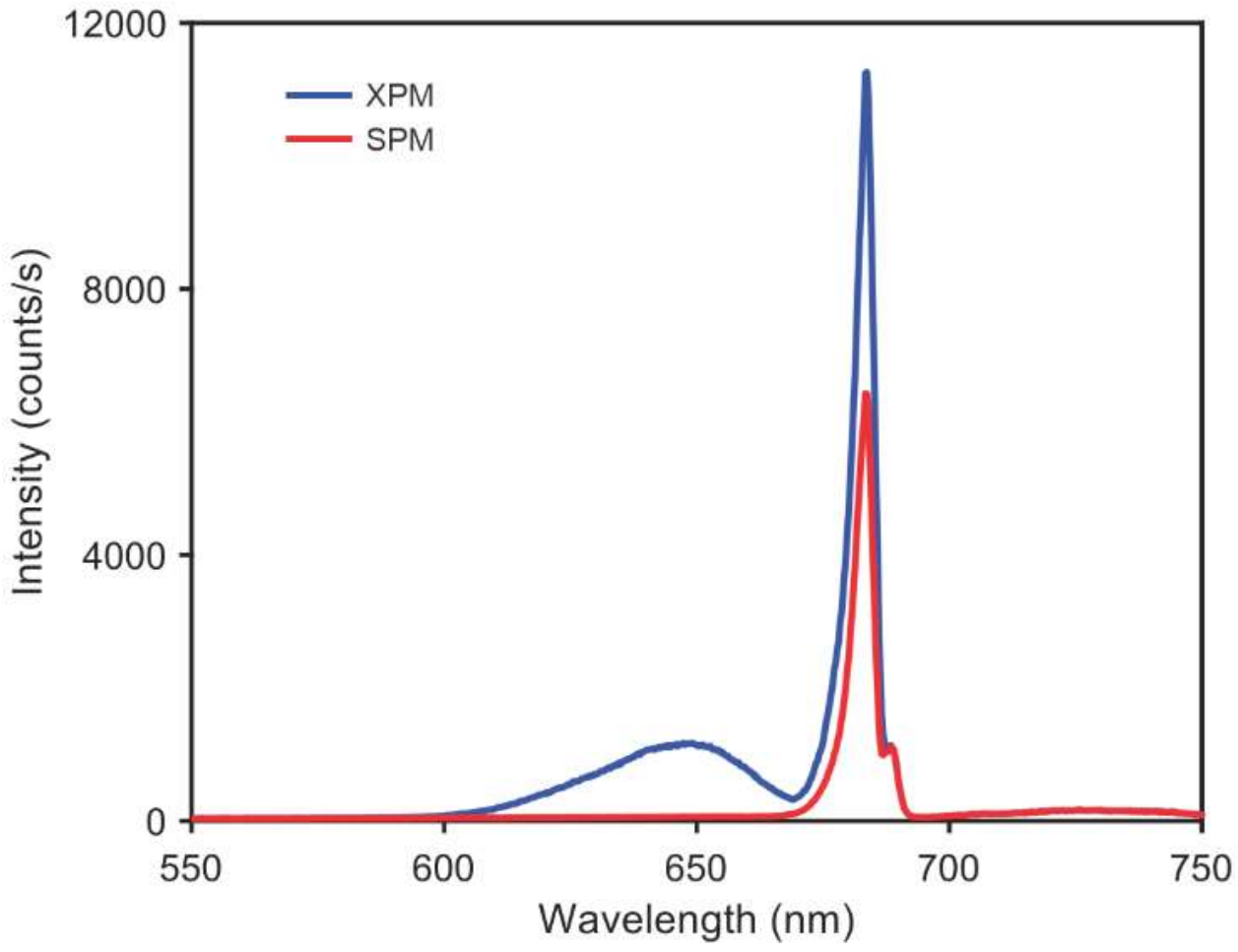

Figure S1. Self-phase modulation (SPM, red curve) and cross-phase modulation (XPM, blue curve) induced spectral broadening of the probe pulse. The spectra were excerpted from the pump-probe measurements shown in Fig. 2a at -1 ps (SPM) and 0 ps (XPM), respectively.

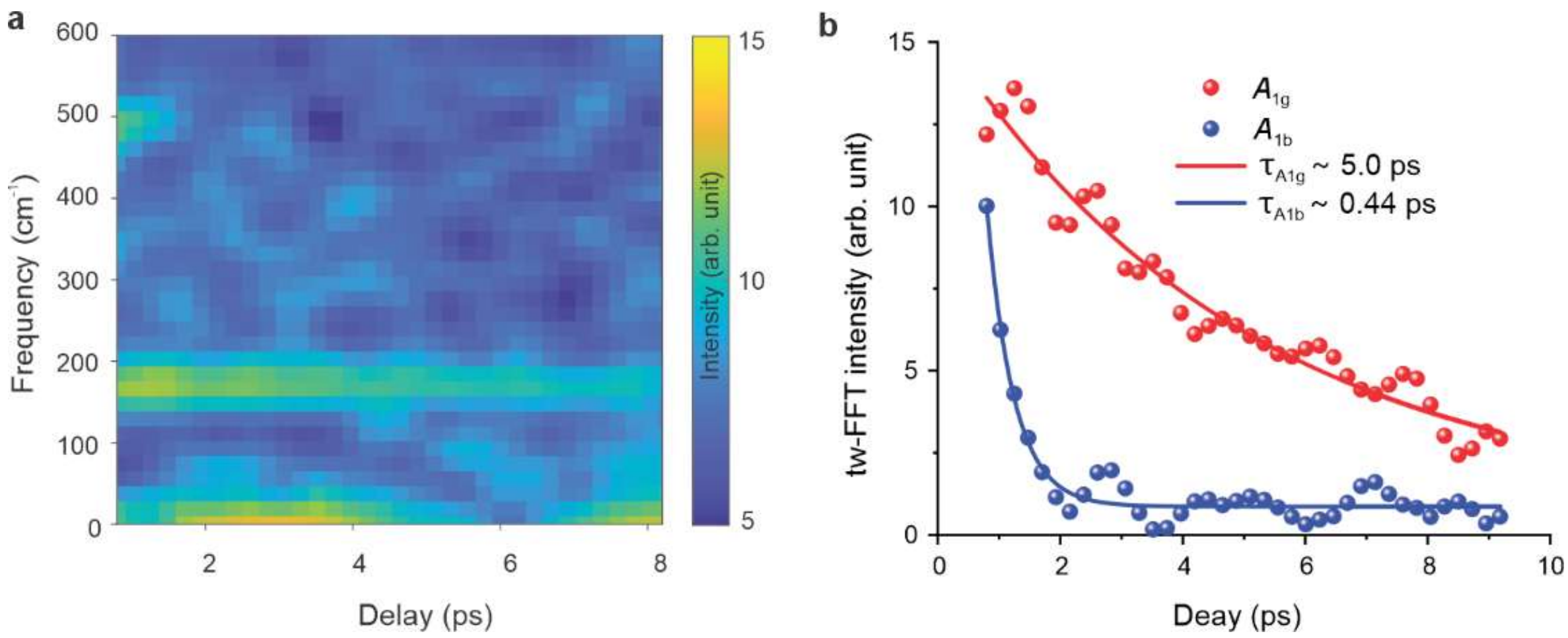

Figure S2. Decay dynamics of the A_{1g} and A_{1b} phonon modes. a, Short-time windowed FFT analysis of the time-domain signal in Fig. 2b from 0.1 ps to 10 ps, using a Kaiser window with a temporal width of ~1.4 ps. **b**, Exponential fits of the phonon amplitudes at 172 cm^{-1} (red) and 480 cm^{-1} (blue) in **a** as a function of the pump-probe delay. The extracted decay constants are ~5.0 ps and ~0.4 ps, respectively.

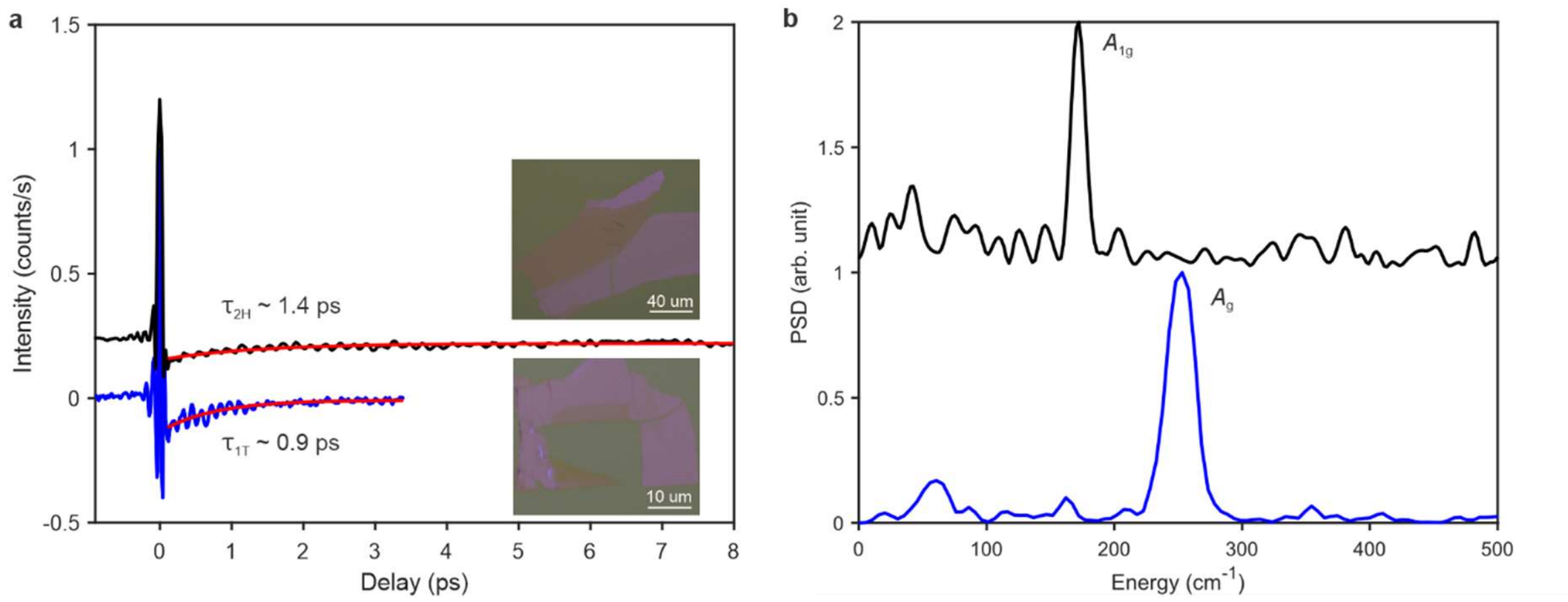

Figure S3. Pump-probe measurements on *2H*-$MoTe_2$ and *1T*-$MoTe_2$ flakes on Si_3N_4 substrate. a, Temporal cross-cuts of the pump-probe spectral measurements at ~680 nm measured on 2*H*-$MoTe_2$ (red curve) and 1*T*-$MoTe_2$ (blue curve) flakes transferred onto a Si_3N_4 substrate. **b**, Fast Fourier transform (FFT) spectra of the time traces shown in **a**, with a time span from 0.1 ps to 6 ps, and from 0.1 ps to 3.5 ps, respectively. Both samples show dominantly the symmetric A_g phonon mode, without the presence of the B_g and E_{2g} modes. This further supports a displacive excitation mechanism for the coherent phonons. Notably, neither sample shows a peak at ~480 cm^{-1}, which further verifies that the corresponding feature observed in Fig. 2c originates from the quartz substrate rather than from any experimental artefact.

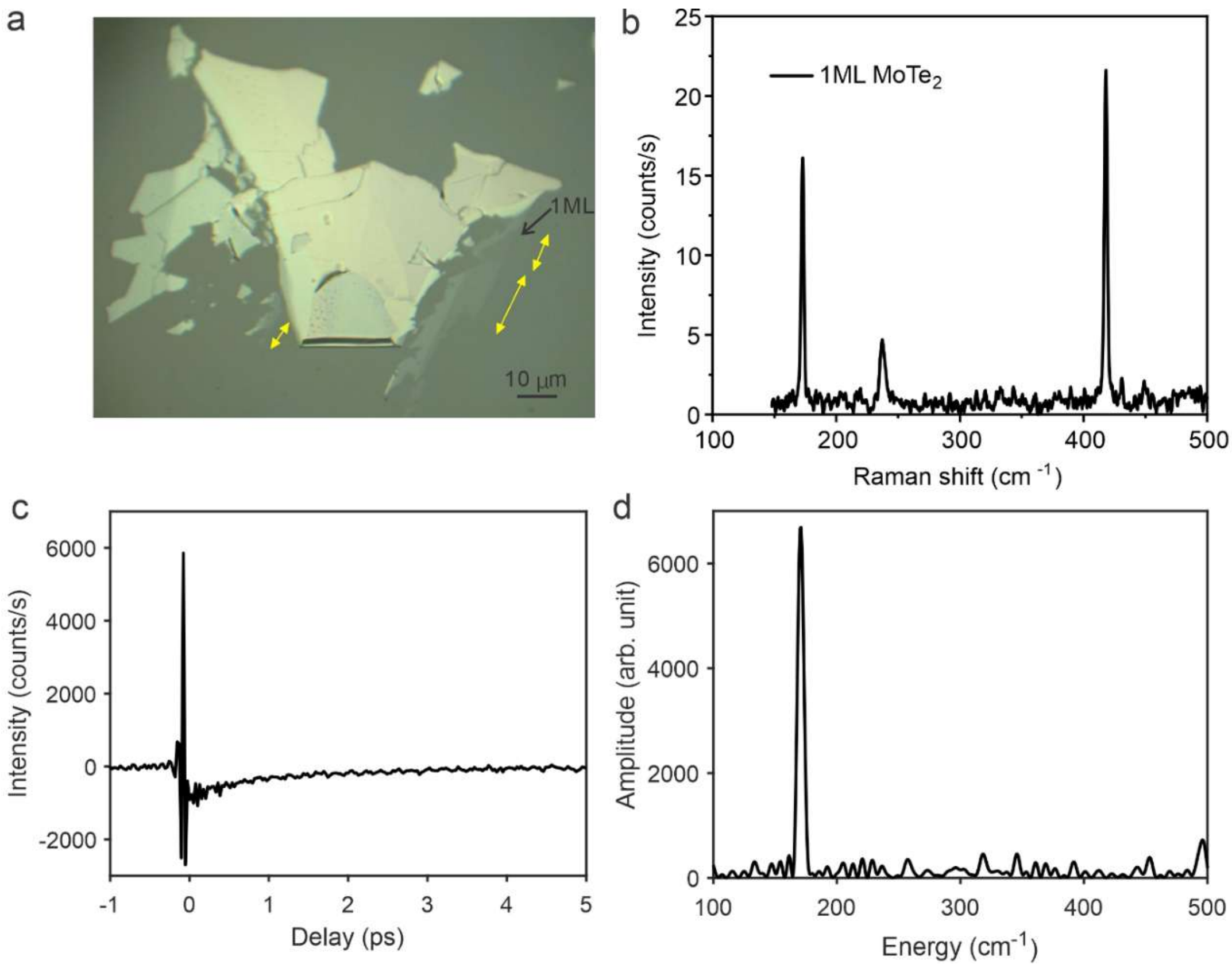

Figure S4. Raman and pump-probe spectroscopy of monolayer $MoTe_2$ on a sapphire substrate. a, Optical micrography of transferred 2*H*-$MoTe_2$ flakes on the sapphire substrate. The monolayer region, indicated by the black arrow, has a lateral size of approximately 10 µm. **b**, Raman spectrum of monolayer $MoTe_2$ measured at the indicated region in **a**. The monolayer thickness is confirmed by the absence of the ~290 cm^{-1} Raman peak.[1,2] The peak at ~418 cm^{-1} originates from the sapphire substrate. A 633 nm continuous-wave laser was used for Raman excitation with a power of 54 µW. **c**, Temporal cross-cut of the XPM spectra at ~680 nm from the pump-probe measurements. Pump and probe laser powers were set to be 2.4 mW and 2.5 mW, respectively. **d**, Fast Fourier transform (FFT) of the time trace in **c**, calculated over a delay range of 0.1 ps to 5 ps. The FFT spectrum reveals a pronounced peak at ~172 cm^{-1}, corresponding to the A_{1g} phonon mode observed in the Raman spectrum in **b**.

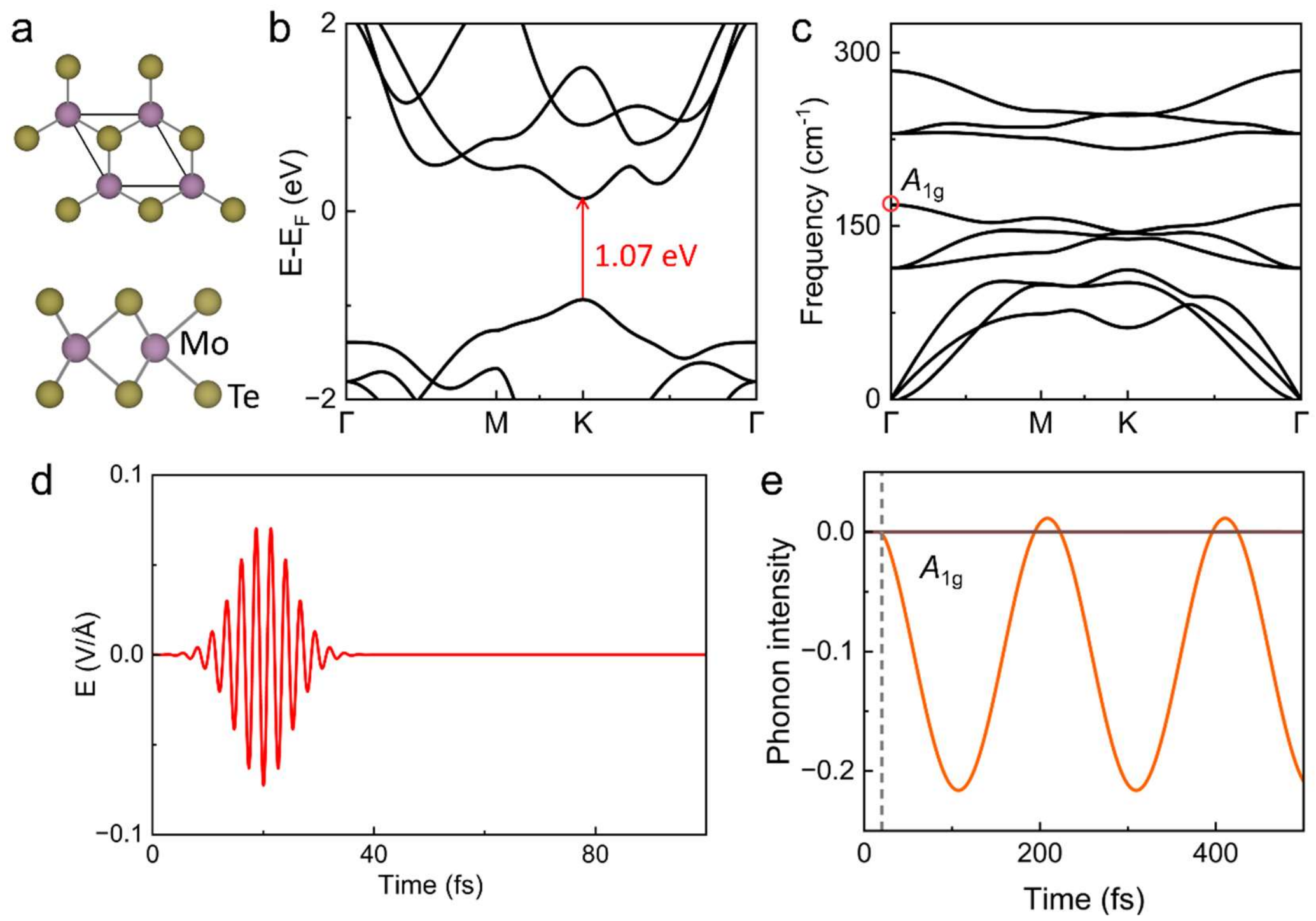

Figure S5. Band structure and displacive excitation of coherent phonons in monolayer 2*H*-MoTe$_2$ evaluated from time-dependent density functional theory (TDDFT) calculations. a, Atomic structure of monolayer of 2*H*-MoTe$_2$ shown in top view (upper panel) and side view (lower panel). **b**, TDDFT calculated band structure of monolayer 2*H*-MoTe$_2$, exhibiting a direct bandgap of 1.07 eV at the *K* point. **c**, Calculated phonon dispersion of monolayer 2*H*-MoTe$_2$, with the symmetric A_{1g} mode located at ~168 cm^{-1} at the Γ point. **d**, Temporal profile of the ultrafast laser pulse used in the TDDFT simulation, $E(t) = E_0\cos(\omega t)\exp\left[\frac{(t-t_0)^2}{2\sigma^2}\right]$, with σ = 5 fs and a photon energy of 1.55 eV, matching with the experimental pump pulse conditions. **e**, Simulated phonon oscillations of the A_{1g} phonon mode induced by the pump pulse. The onset of the optical excitation is indicated by the dashed line.

I. Four-wave mixing (FWM) and coherent anti-Stokes Raman scattering (CARS) processes

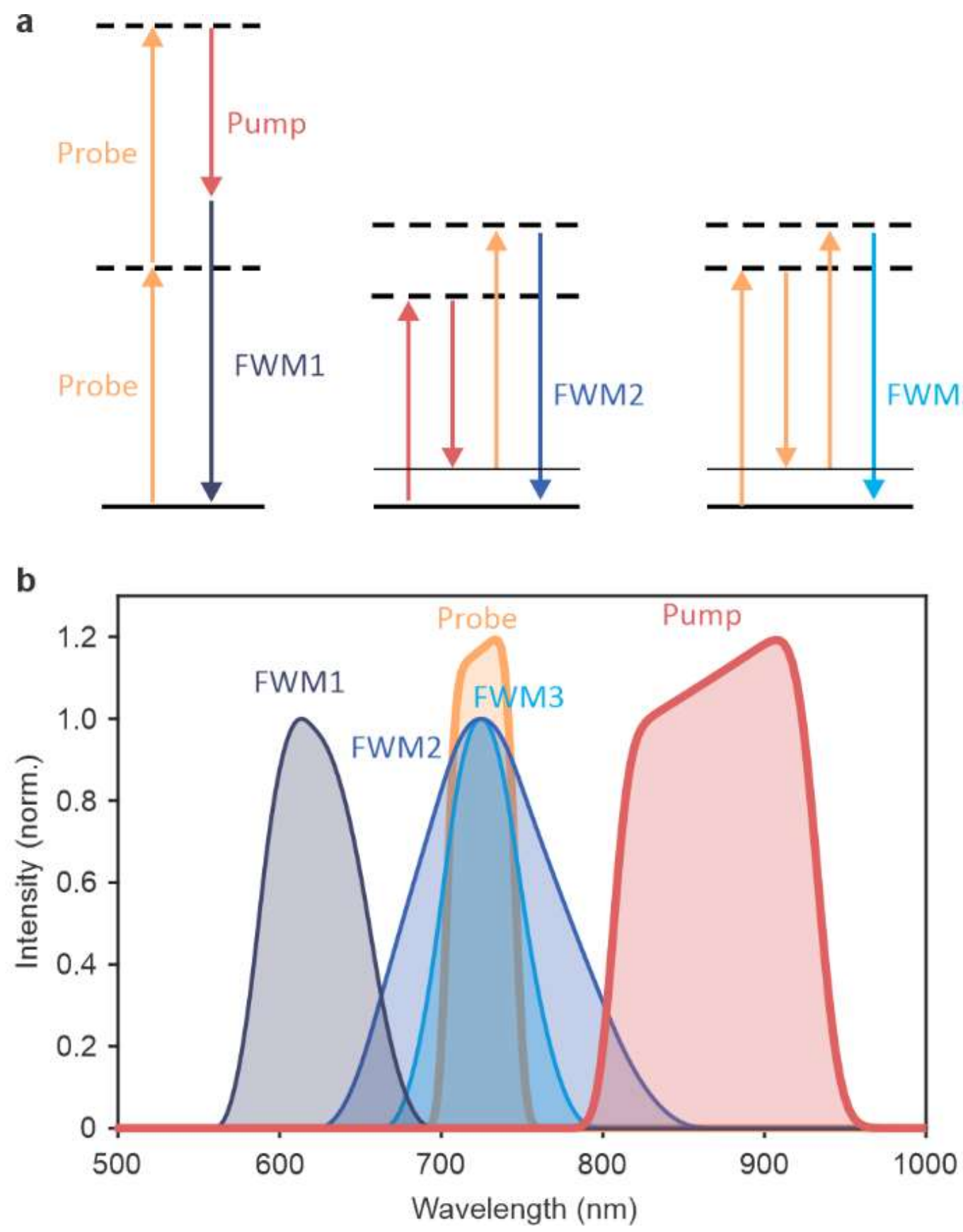

Figure S6. Four-wave mixing processes at the zero time delay between pump and probe pulses. a, Schematic illustration of the relevant FWM processes when the pump and probe pulses temporally overlap. Process 1 (FWM1): involving two photons from the probe pulse and one photon from the pump pulse. Process 2 (FWM2): involving two photons from the pump pulse and one photon from the probe pulse. Process 3 (FWM3): degenerate four-wave mixing generated solely by the probe pulse, which is usually much lower in efficiency compared to the processes 1 and 2. **b**, Normalized spectra of the pump, probe, and resulting FWM signals, highlighting the spectral separation and wavelength region below 700 nm where the FWM contributions are observed.

In principle, FWM processes 1 and 2 both could generate vibrational coherence, in close analogy to the case of coherent anti-Stokes Raman scattering (CARS).[3–5] However, these processes typically require high pulse energies and strict phase matching conditions. In our collinear

geometry, such conditions are not well stratified since a low numerical aperture objective (NA = 0.5) is used in the measurement. Moreover, in our experiment configuration, any anti-Stokes Raman scattering signal (FWM2) of the A_{1g} mode (corresponding to a Raman shift of 172 cm^{-1} from the probe pulse, i.e., from 692 nm to 740 nm in wavelength) generated by the probe pulse (bandwidth from 700 nm to 750 nm) would be fully suppressed by the 690 nm shortpass filter placed before the spectrometer, see Fig. 1 in main-text. Thus, the detected signal at ~ 680 nm is dominated by cross-phase modulation (XPM) near the blue spectral edge of the probe pulse. The observed quadratic dependence of signal intensity at ~680 nm on probe fluence (Fig. 3a) further rules out the CARS (FWM2) as the origin of the induced vibrational coherence.

Compared with conventional FWM based spectroscopies, such as impulsive stimulated Raman spectroscopy (ISRS) and coherent anti-Stokes Raman scattering, which rely on resonant interactions and high pulse energies,[6–8] the present XPM-based approach offers several distinct advantages. In particular, it eliminates the requirement for a third Raman pulse and strict phase-matching conditions, while remaining sensitive to low-frequency vibrational modes. These features make XPM a robust and low-power alternative for probing coherent lattice dynamics in quantum materials.

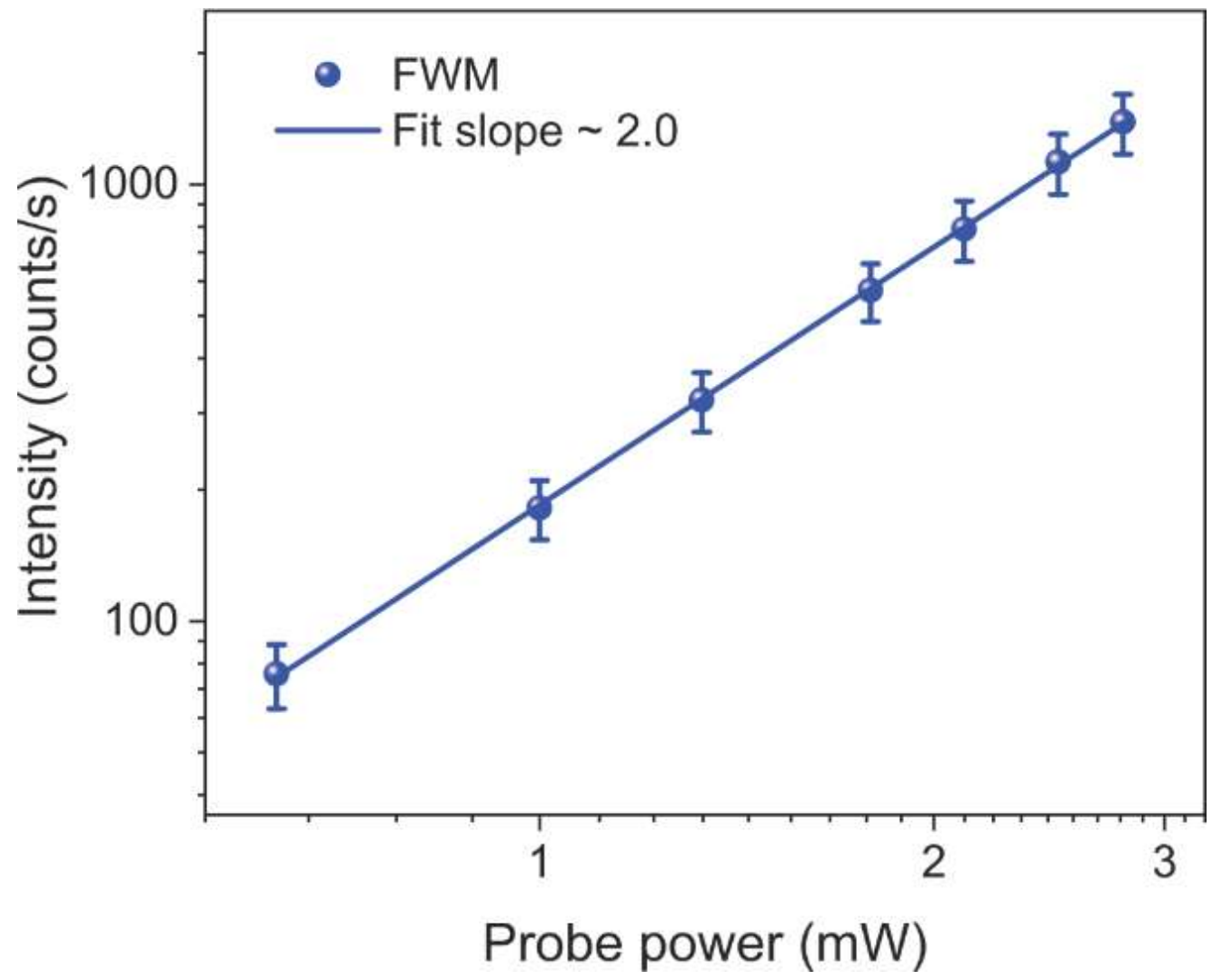

Figure S7. Fluence dependence of the FWM signal. Variation of the intensity of FWM signal (process 1) recorded at ~650 nm, corresponding to a FWM process involving two photons from the probe pulse and one photon from the pump pulse, as a function of the increasing fluence of the probe pulse. The signal follows the expected quadratic scaling with probe power. Blue curve shows the quadratic fitting in the dual logarithmic axes, with fit parameters annotated in the legend.

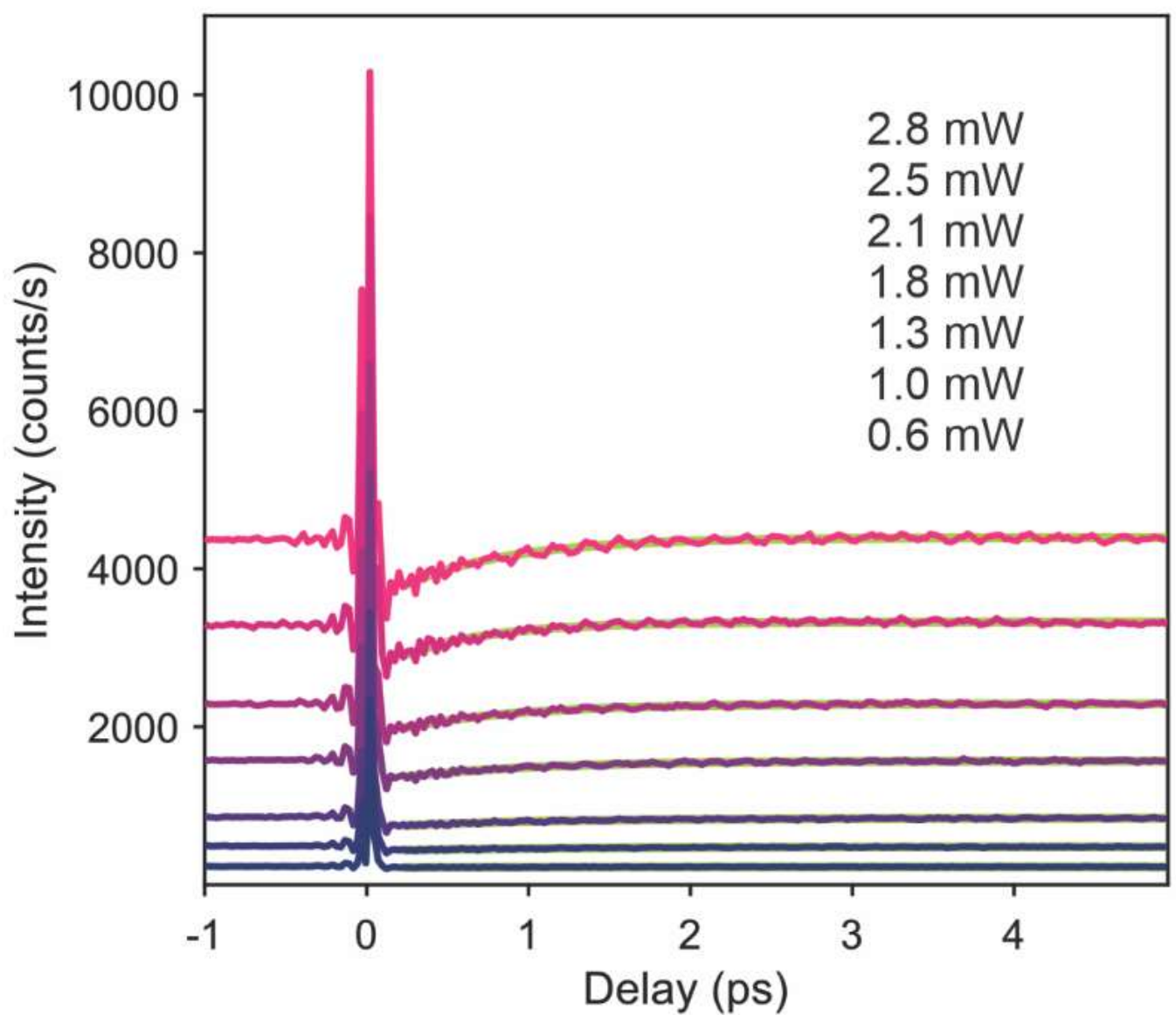

Figure S8. A series of temporal cross-cuts retrieved from spectral pump-probe measurements at ~680 nm with increasing fluence of the probe pulses, with the fluence of the pump pulses fixed at

2.2 mW. The corresponding probe fluence values are annotated sequentially in order of the acquisition (from bottom to top). An acquisition time of 0.5 s per delay step was used for all the spectra.

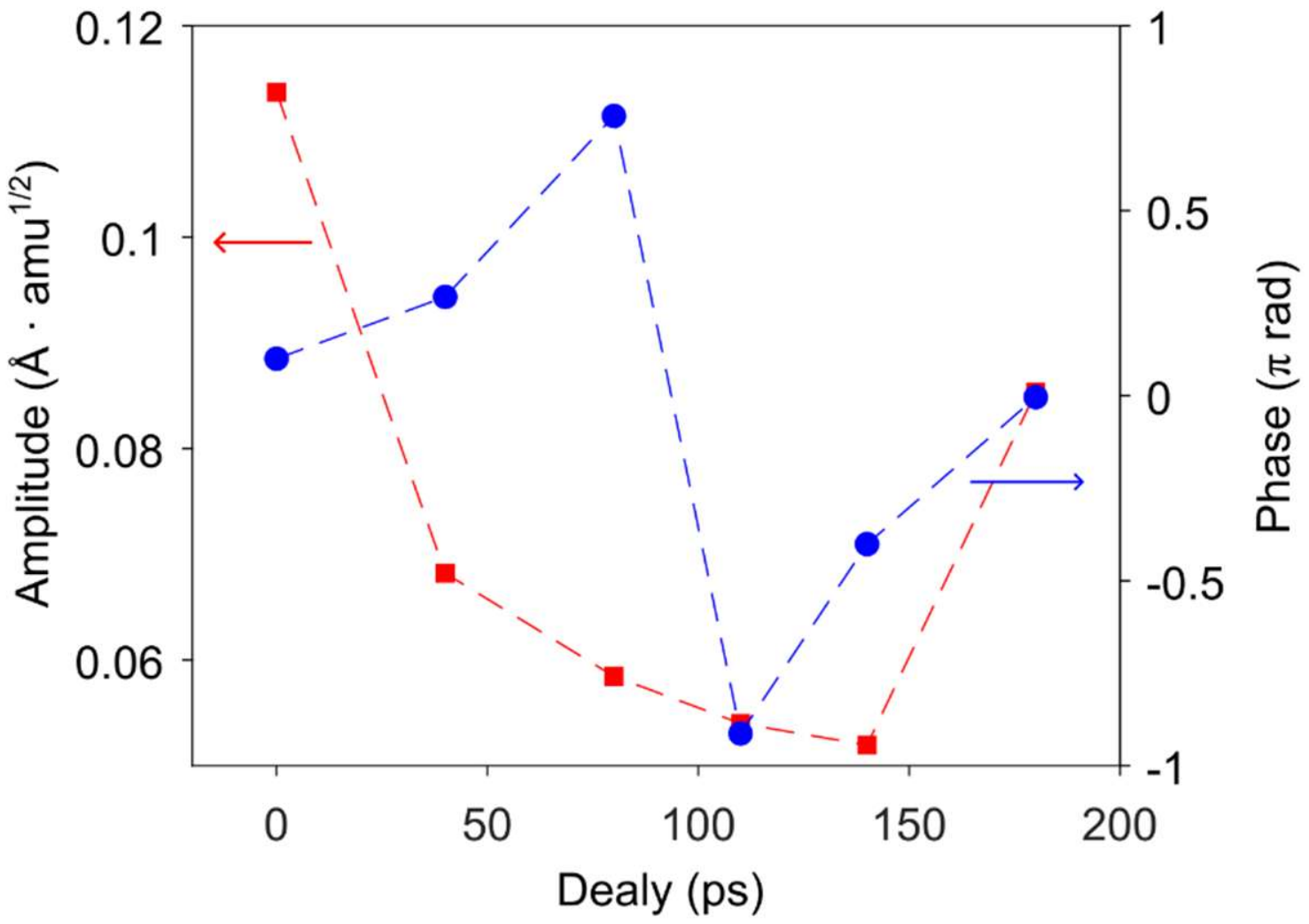

Figure S9. TDDFT calculated amplitude and phases of phonon oscillations under dual-pump pulse excitation. Calculated phonon oscillation amplitudes (red squares) and phases (blue circles) as a function of the delay between the two excitation pulses. The phonon amplitude reaches a maximum when the two pump pulses temporally overlap and the oscillation phase is close to zero. These results show good agreement with the experimental data presented in Fig. 4f of the main-text.

Table S1. Comparison of the present XPM approach with representative studies of Kerr nonlinearity and ultrafast spectroscopy.

Study	Material/ method	Excitation condition	Characteristic response	Detection/ modulation	Mechanism
Present work	Few layer 2H-$MoTe_2$ / Phase-sensitive XPM	6-38 pJ (~60-380 µJ/cm^2); pump ~10 fs, probe ~20 fs; 80 MHz	Electronic: sub-ps; A_{1g} phonon coherence ~5 ps	Time-dependent spectral redistribution and COM shift, 2.5%	Carrier redistribution and coherent phonon modulation in the third-order susceptibility
M. Zhang *et al.*, Phys. Rev. X 9, 021036 (2019)[9]	T_d-$MoTe_2$ single crystal / Linear transient reflectivity	Pump fluence 100 µJ/cm^2 - 5 mJ/cm^2; ~35 fs; 1 kHz	Fast response ~0.3 ps; slow response hundreds of ps	ΔR/R with a lock-in technique, ~0.1%	DECP and light-induced structural symmetry change
C. Sayers *et al.*, Nano Lett. 23, 9235 (2023)[10]	Monolayer $MoTe_2$ / Broadband transient reflection	Pump fluence ~500 µJ/cm^2; ~40 fs	Electronic decay ~0.8 ps and ~6-8 ps coherent phonons	ΔR/R with a lock-in technique, ~1%	DECP and phonon-exciton coupling
X. Zheng *et al.*, Opt. Express 23, 15616–15623 (2015)[11]	Monolayer WS_2 / Z-scan	~1.38 µJ; ~100 fs; 1 kHz	Not time-resolved	n_2 ~8.1×10^{-13} m^2/W	Kerr nonlinear refraction with free-carrier contribution
H. Qian et al., Nat. Commun. 7, 13153 (2016)[12]	3 nm Au quantum well / Z-scan and SPM	~1.4-8.6 nJ; 80 fs; 80 MHz	Not time-resolved	Spectral broadening, n_2 ~3.9×10^{-13} m^2/W	Quantum well enhanced Kerr nonlinearity
J. Zhang *et al.*, Nat. Photon. 18, 792–798 (2024)[13]	z-cut α-quartz crystal / High-harmonic spectroscopy	Pump ~300 mJ/cm^2, Probe ~25 mJ/cm^2; ~25-30 fs	Femtosecond phonon dynamics	Energy modulation ~14-60 cm^{-1}	e-ph and anharmonic ph-ph coupling

References

1. Ruppert, C., Aslan, O. B. & Heinz, T. F. Optical properties and band gap of single- and few-layer MoTe2 crystals. *Nano Lett.* **14**, 6231–6236 (2014).
2. Park, J., Kim, Y., Jhon, Y. I. & Jhon, Y. M. Temperature dependent Raman spectroscopic study of mono-, bi-, and tri-layer molybdenum ditelluride. *Appl. Phys. Lett.* **107**, 153106 (2015).

3. Luo, Y. *et al.* Imaging and controlling coherent phonon wave packets in single graphene nanoribbons. *Nat. Commun.* **14**, 3784 (2023).
4. Cheng, J.-X., Volkmer, A. & Xie, X. S. Theoretical and experimental characterization of coherent anti-Stokes Raman scattering microscopy. *J. Opt. Soc. Am. B* **19**, 1363 (2002).
5. Yampolsky, S. *et al.* Seeing a single molecule vibrate through time-resolved coherent anti-Stokes Raman scattering. *Nat. Photonics* **8**, 650–656 (2014).
6. Raanan, D., Ren, L., Oron, D. & Silberberg, Y. Impulsive Raman spectroscopy via precision measurement of frequency shift with low energy excitation. *Opt. Lett.* **43**, 470 (2018).
7. Kukura, P., McCamant, D. W. & Mathies, R. A. Femtosecond stimulated Raman spectroscopy. *Annu. Rev. Phys. Chem.* **58**, 461–488 (2007).
8. Kuramochi, H. & Tahara, T. Tracking Ultrafast Structural Dynamics by Time-Domain Raman Spectroscopy. *J. Am. Chem. Soc.* **143**, 9699–9717 (2021).
9. Zhang, M. Y. *et al.* Light-Induced Subpicosecond Lattice Symmetry Switch in MoTe2. *Phys. Rev. X* **9**, 021036 (2019).
10. Sayers, C. J. *et al.* Strong Coupling of Coherent Phonons to Excitons in Semiconducting Monolayer MoTe2. *Nano Lett.* **23**, 9235–9242 (2023).
11. Zheng, X., Zhang, Y., Chen, R., Cheng, X. & Xu, Z. Z-scan measurement of the nonlinear refractive index of monolayer WS2. *Opt. Lett.* **23**, 15616–15623 (2015).
12. Qian, H., Xiao, Y. & Liu, Z. Giant Kerr response of ultrathin gold films from quantum size effect. *Nat. Commun.* **7**, 1–6 (2016).
13. Zhang, J. *et al.* High-harmonic spectroscopy probes lattice dynamics. *Nat. Photonics* **18**, 792–798 (2024).
